# Inhibition of Pannexin 1 Reduces the Tumorigenic Properties of Human Melanoma Cells

**DOI:** 10.3390/cancers11010102

**Published:** 2019-01-16

**Authors:** Taylor J. Freeman, Samar Sayedyahossein, Danielle Johnston, Rafael E. Sanchez-Pupo, Brooke O’Donnell, Kenneth Huang, Zameena Lakhani, Daniel Nouri-Nejad, Kevin J. Barr, Luke Harland, Steven Latosinsky, Aaron Grant, Lina Dagnino, Silvia Penuela

**Affiliations:** 1 Department of Anatomy and Cell Biology, Schulich School of Medicine & Dentistry, University of Western Ontario, London, ON N6A5C1, Canada; tayfreem3@gmail.com (T.J.F.); ssayedy@uwo.ca (S.S.); Danielle.Johnston@schulich.uwo.ca (D.J.); rsnchezp@uwo.ca (R.E.S.-P.); bodonne3@uwo.ca (B.O’D.); khuang85@uwo.ca (K.H.); zlakhan3@uwo.ca (Z.L.); dnourin@uwo.ca (D.N.-N.); kevin.barr@schulich.uwo.ca (K.J.B.); luke.harland@trinity.ox.ac.uk (L.H.); 2 Surgery, Schulich School of Medicine & Dentistry, University of Western Ontario, London, ON N6A5C1, Canada; steven.latosinsky@lhsc.on.ca (S.L.); Aaron.Grant@lhsc.on.ca (A.G.); 3 Physiology and Pharmacology, Schulich School of Medicine & Dentistry, University of Western Ontario, London, ON N6A5C1, Canada; ldagnino@uwo.ca; 4 Oncology, Schulich School of Medicine & Dentistry, University of Western Ontario, London, ON N6A5C1, Canada

**Keywords:** pannexin, PANX1, melanoma, carbenoxolone, probenecid, tumor growth, patient-derived cells, Chick-CAM, xenografts, ATP, Wnt, β-catenin

## Abstract

Pannexin 1 (PANX1) is a channel-forming glycoprotein expressed in many tissues including the skin. PANX1 channels allow the passage of ions and molecules up to 1 kDa, including ATP and other metabolites. In this study, we show that PANX1 is highly expressed in human melanoma tumors at all stages of disease progression, as well as in patient-derived cells and established melanoma cell lines. Reducing PANX1 protein levels using shRNA or inhibiting channel function with the channel blockers, carbenoxolone (CBX) and probenecid (PBN), significantly decreased cell growth and migration, and increased melanin production in A375-P and A375-MA2 cell lines. Further, treatment of A375-MA2 tumors in chicken embryo xenografts with CBX or PBN significantly reduced melanoma tumor weight and invasiveness. Blocking PANX1 channels with PBN reduced ATP release in A375-P cells, suggesting a potential role for PANX1 in purinergic signaling of melanoma cells. In addition, cell-surface biotinylation assays indicate that there is an intracellular pool of PANX1 in melanoma cells. PANX1 likely modulates signaling through the Wnt/β-catenin pathway, because β-catenin levels were significantly decreased upon PANX1 silencing. Collectively, our findings identify a role for PANX1 in controlling growth and tumorigenic properties of melanoma cells contributing to signaling pathways that modulate melanoma progression.

## 1. Introduction

Pannexins (Panx1, Panx2, Panx3) are a family of glycoproteins [1,2,3,4] that form large-pore channels at the cell membrane [4,5]. Panx1 is widely expressed in many cells and organs, while Panx2 and Panx3 have more restricted expression patterns [6]. Pannexin 1 channels (Panx1 in rodents, or PANX1 in humans) allow the passage of molecules up to 1 kDa in size important for paracrine and autocrine signaling, including adenosine triphosphate (ATP), which they release at the cell surface [1,4,7,8]. Panx1 channels have also been reported to function intracellularly as a calcium leak channel in the endoplasmic reticulum [9,10].

PANX1 is involved in multiple disease states, including cancer [11]. For most cancer types, Panx1 expression positively correlates with the onset or progression of the disease [12]. For example, human leukemic lymphocytes show higher levels of PANX1 in comparison to normal T cells, and high expression of PANX1 has been reported across aggressive multiple myeloma cell lines [13,14]. A study demonstrated high PANX1 mRNA expression in human glioma cell lines. However, the authors also found that rat C6 glioma cells lacked Panx1, and upon overexpressing green fluorescent protein (EGFP) -tagged Panx1 they observed a reduction in several cancerous properties of the rat cells, as well as increased gap junction communication. These contradictory results could have been produced by the effect of Panx1-GFP overexpression on endogenous connexins, or potential differences in the role of Panx1 between species [15]. Furthermore, a significant reduction in proliferation of U87-MG human glioma cells was noted when a PANX1 siRNA was introduced [16]. Reduced levels of PANX1 and PANX3 in basal cell and squamous cell carcinomas compared to normal epidermal tissue supports the idea that the role of PANX1 may also differ between cancer subtypes [17]. In breast cancer cells, the co-expression of a truncated mutant form (PANX1^1–89^) with the full-length form of PANX1 led to augmented ATP release. ATP acts on P2Y-purinergic receptors, resulting in resistance to apoptosis and enhanced cell survival in distant organ metastases. This effect was reduced with PANX1 channel blockers [18].

Two FDA- and Health Canada-approved drugs, carbenoxolone (CBX) and probenecid (PBN), are commonly used to block Panx1 channels [19,20] and have been used in different applications to attenuate release of ATP and other metabolites [18,21,22]. Probenecid was originally used for the treatment of gout [20] and carbenoxolone for peptic ulcer [19]. Both drugs can permeate into the cells, reaching Panx1 at either the cell surface or in intracellular compartments, and act as Panx1 channel blockers. The blockers have been shown to interact with the first extracellular loop of the Panx1 protein to inhibit channel function [23]. Panx1 channel blockers, CBX and PBN also attenuate pathological changes, including neurodegeneration after ischemic injury and irregular gut motility in inflammatory bowel diseases [20,23,24,25,26,27]. In addition, pre-treatment with CBX significantly reduced the ability of highly aggressive breast cancer cells to metastasize to the lungs in a mouse model of this disease [18].

Melanoma is a highly aggressive tumor type, causally associated with over 75% of skin cancer deaths, and increasing in incidence every year [28,29]. A primary melanoma tumor develops from the malignant transformation of melanocytes [30,31]. Cells from the primary tumor can metastasize through lymphatic vessels forming nodal tumor metastases and/or further spread throughout the vascular system to colonize distant organs including the brain, liver and lungs [30,32]. Current treatments for melanoma that focus primarily on inhibiting proteins of altered signaling pathways present in a subset of affected individuals (i.e., BRAF-targeted therapy) [33,34,35] frequently result in the development of resistance to therapy [36,37]. Despite recent advances using checkpoint inhibitors [38,39], up to 70% of metastatic melanoma patients still do not respond to immunotherapies [40]. Therefore, a better understanding of the cellular mechanisms that drive melanoma progression is required to find additional therapeutic alternatives. We previously reported that Panx1 expression was significantly up-regulated in B16-BL6 mouse melanoma cells compared to normal melanocytes. By using shRNA knockdown to reduce Panx1 levels in B16-BL6 cells, we observed a significant reduction in their malignant properties including decreased proliferation, migration and tumor size, as well as development of fewer metastases in the liver of a chick embryo model [17].

In the current study, we identified high levels of endogenous PANX1 protein in human melanoma cell lines, patient biopsies and patient-derived melanoma cells from primary, nodal and distant tumors. Using PANX1 channel blockers as well as shRNA vectors to reduce PANX1 function, we were able to reduce melanoma cell growth and migration, and significantly decrease primary tumor growth in a xenograft model. Reduced ATP release and β-catenin levels were apparent when PANX1 was blocked or silenced, respectively, indicating different signaling mechanisms regulated by PANX1. Our results suggest that high levels of PANX1 found in human melanomas at different stages of disease progression may be associated with their malignant behavior, making PANX1 a potential new target for therapeutic intervention.

## 2. Results

### 2.1. Pannexin 1 Is Expressed in Patient-Derived Primary Melanoma Tumors, as Well Nodal and Distant Melanoma Metastases

**Figure 1:**
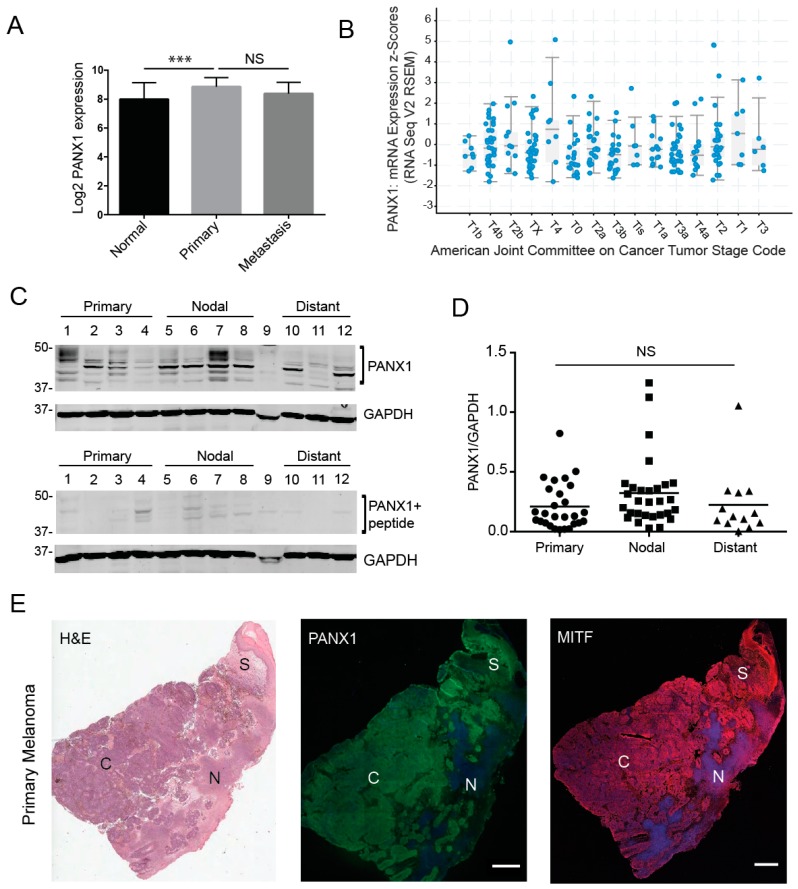
Pannexin 1 is expressed in patient-derived primary melanoma tumors, as well nodal and distant melanoma metastases. (**A**) Gene expression analyses from microarray study GSE15605 revealed significantly higher PANX1 expression in primary human melanoma tumors relative to normal skin biopsies. (**B**) Analysis of PANX1 mRNA expression in melanoma tumors of the Cancer Genome Atlas (TCGA) database revealed that PANX1 expression is not significantly different across melanoma stages (denoted according to the Cancer Tumor Stage Code). (**C**) Representative PANX1 protein levels in patient-derived melanoma tumors. Numbers represent different patient donors. A peptide pre-absorption assay confirms antibody specificity. Protein sizes in kDa. (**D**) Densitometry was used to quantify PANX1 levels (bands in bracket) in all primary, nodal and distant melanoma samples provided by the Ontario Institute for Cancer Research (OICR), normalized to GADPH (Sample 9 was degraded and not used for analysis). There is no significant difference in PANX1 levels among stages of melanoma progression. Primary tumors (N = 6 patients, n = 27 technical reps), Nodal mets (N = 7, n = 29), Distant mets (N = 3, n = 13); One-way ANOVA followed by Tukey’s *post-hoc* test was used to analyze data. line = mean; NS = not significant. (**E**) Patient-derived primary melanoma tumor labeled with PANX1 (green). Sequential sections of the tumor stained using H&E (provided by OICR) and a marker for a melanocytic-lineage, MITF (red). Melanoma core (C), Necrotic regions of the tumor (N), Stromal area of the tumor (S). Bar: 1000 μm.

To investigate whether PANX1 plays a role in the context of human melanoma, we first evaluated gene expression analyses in a microarray study [41,42]. The data showed that PANX1 expression in primary melanoma tumors is significantly increased relative to normal skin biopsies (Figure 1A). It should be noted that skin biopsies and melanoma samples also contain other cell types in addition to the normal or transformed melanocytes. We also found that there were no significant differences in PANX1 mRNA expression between cutaneous melanoma tumors at different stages in the Cancer Genome Atlas (TCGA) database [43,44] (Figure 1B). Next, we examined if PANX1 protein was present in melanoma biopsies. Using our previously characterized antibodies [3,45], PANX1 was readily detected in primary melanoma tumors, nodal and distant metastases obtained from banked specimens (Figure 1C–E, Supplementary Figure S1). Immunoblot analysis revealed that all tumor samples were positive for PANX1 expression, with the characteristic banding pattern (Figure 1C) [45]. PANX1 exhibits a multi-banding pattern in immunoblots due to different levels of N-glycosylation [3,46]. We observed high variation in PANX1 levels amongst individuals, but no significant difference was observed in PANX1 among the different stages of melanoma progression in the samples examined (Figure 1D). We combined immunostaining for PANX1, and the melanocytic-lineage marker to identify melanoma tumor cells, Microphthalmia/Melanogenesis-associated transcription factor (MITF) [47] with sequentially sectioned biopsies staining using hematoxylin and eosin (H&E). PANX1 staining was found primarily in melanoma tumor cores and stromal regions (Figure 1E), with no labeling found in necrotic areas. Immunofluorescence microscopy analysis from all melanoma biopsies examined was consistent with our immunoblot data, demonstrating individual variation in PANX1 levels (Supplementary Figure S1).

### 2.2. PANX1 Is Highly Expressed in Patient-Derived Primary Melanoma Cells

**Figure 2:**
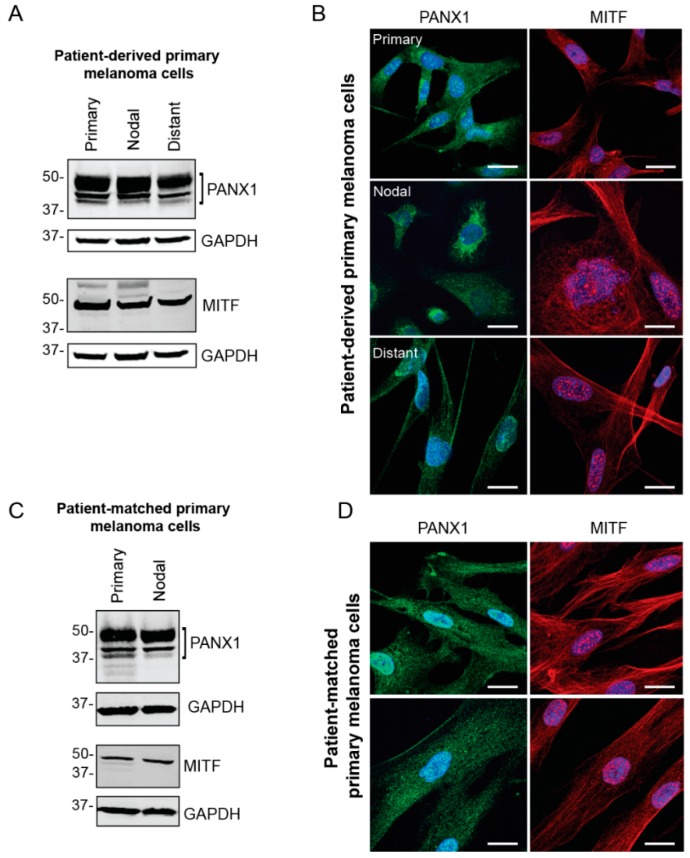
PANX1 is highly expressed in patient-derived primary melanoma cells. (**A**) Representative PANX1 levels in primary cells derived from melanoma biopsies of patient tumors with primary (N = 5), nodal (N = 4) and distant (N = 4) metastases. Cultures of primary melanoma cells were distinguished through MITF expression. (**B**) Patient-derived primary melanoma cells extracted from three stages of melanoma progression express PANX1 intracellularly and at the cell membrane. MITF is a transcription factor involved in melanocytic lineages and is found in the nucleus and in the cytoplasm of the cell. (**C**) Patient-matched primary cells were extracted from a primary tumor and a nodal metastasis within a single patient and show high PANX1 levels. Melanoma identity was confirmed with MITF expression. (**D**) Patient-matched primary cells immunolabeled for PANX1 show intracellular and cell membrane localization. PANX1: green, MITF: red, Hoechst: blue; Bar: 20 μm.

Primary cells were extracted and cultured from fresh surgical specimens obtained from local melanoma surgeries performed at the London Health Sciences Centre (LHSC) Canada. Cells were derived from fresh primary, nodal and distant melanoma tumors to evaluate PANX1 levels and localization in the melanoma cells from each tumor. To assess the identity of primary melanoma cell cultures, the presence of MITF was examined via western blotting and immunofluorescence microscopy (Figure 2). Our results show high endogenous PANX1 levels in primary cells derived from three different stages of melanoma progression compared between patients (Figure 2A), or among stages of progression in the same patient (Figure 2C). PANX1 was localized mostly intracellularly, but we also found evidence of labeling at the cell surface of primary melanoma cells (Figure 2B,D). Taken together, this sampling of human melanoma biopsies and patient-derived primary cells indicates that PANX1 is present at high levels in melanoma tumors and cells, and at all stages of melanoma progression.

### 2.3. Pannexin 1 Is Expressed in Established Isogenic Human Melanoma Cell Lines

**Figure 3:**
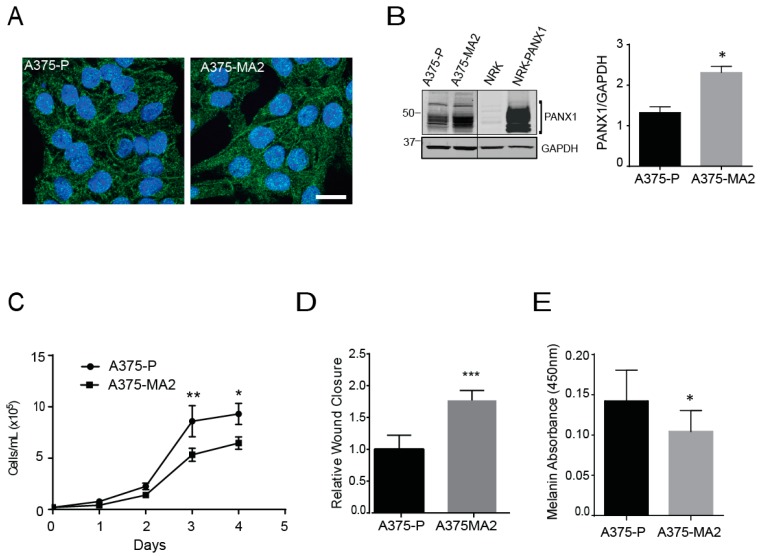
Pannexin 1 is expressed in established human melanoma cell lines. (**A**) A375-P and A375-MA2 human melanoma cells were fixed and stained with anti-PANX1 polyclonal antibodies (green). DNA was stained with Hoechst (blue). Bar: 20 μm. Representative fields are shown from three separate experiments. (**B**) A375-P, A375-MA2, Normal Rat Kidney cells (NRK), and NRK overexpressing PANX1 (NRK-PANX1) cells were lysed and equal amounts of protein were resolved by SDS-PAGE. The blots were probed with the anti-PANX1 antibodies. Black line denotes that both images are from the same blot but scanned with different intensity. Right panel shows the quantification of PANX1 bands in A375-P and A375-MA2 samples using ImageStudio 3.0. The data are expressed as means ±SEM (N = 4) Statistical analyses using student’s *t*-tests; * *p* < 0.05. (**C**) A375-P and A375-MA2 melanoma cells were cultured at 20,000 cells per well in medium containing 10% FBS. Cells were counted daily up to 4 days after initial seeding of the cells onto the 24-well culture dish. The data are expressed as mean ± SEM (N = 5, n = 25). Two-way ANOVA was used to statistically analyze data. * *p* < 0.05, ** *p* < 0.01. (**D**) Scratch assays were performed on a confluent monolayer of A375-P and A375-MA2 cells to assess migration. Quantifications represents cell migration three days following the initial scratch and treatment application. Statistical analysis was performed using student’s *t*-tests; *** *p* < 0.001. (**E**) Melanin was extracted from one million A375-P and A375-MA2 cells. There was significantly decreased melanin content detected in A375-MA2 compared to A375-P cells at the same passage number. Statistical analyses for melanin extraction data includes student’s *t*-tests; * *p* < 0.05.

Given the limited nature and shorter lifespan of primary cells from patients, we set out to evaluate the endogenous PANX1 expression in a panel of established human melanoma cell lines that differ in origin and metastatic profiles. From this survey, we selected two cell lines: A375-P and A375-MA2 melanoma lines that are isogenic lines from A375 cells, and which are excellent cell models of this disease [48]. However, these two lines are quite different since A375-P cells are poorly metastatic, whereas the aggressive A375-MA2 was derived from two selections of A375 lung metastases in immunodeficient mice [49]. Immunofluorescence analysis revealed PANX1 is localized intracellularly and at the cell surface of both human melanoma cell lines (Figure 3A), comparable to our patient-derived primary cells (Figure 2B), with apparent punctate staining in some cells. We also observed increased PANX1 abundance in A375-MA2 compared to A375-P melanoma cells, (Figure 3A,B). Normal rat kidney (NRK) cells with low expression of PANX1 were used as a negative control and exogenous overexpression of PANX1 in NRK was used as a positive control in this experiment (Figure 3B). Next, we analyzed the proliferation characteristics of A375-P and A375-MA2 melanoma cell lines and we observed that A375-MA2 cells in culture show about 32% lower cell numbers at days three and four post-plating, compared to A375-P cells (Figure 3C). In contrast, A375-MA2 cells exhibit about a 1.7 (±0.2)-fold increase in their migratory capacity compared to A375-P (Figure 3D), as revealed in scratch-wound assays. These findings are consistent with the metastatic characteristics reported in vivo for A375-P and A375-MA2 [49]. In addition, A375-MA2 cells showed slightly lower melanin content compared to A375-P cells (Figure 3E).

### 2.4. Silencing of PANX1 Reduces Growth and Migration in Human Melanoma Cells In Vitro

**Figure 4:**
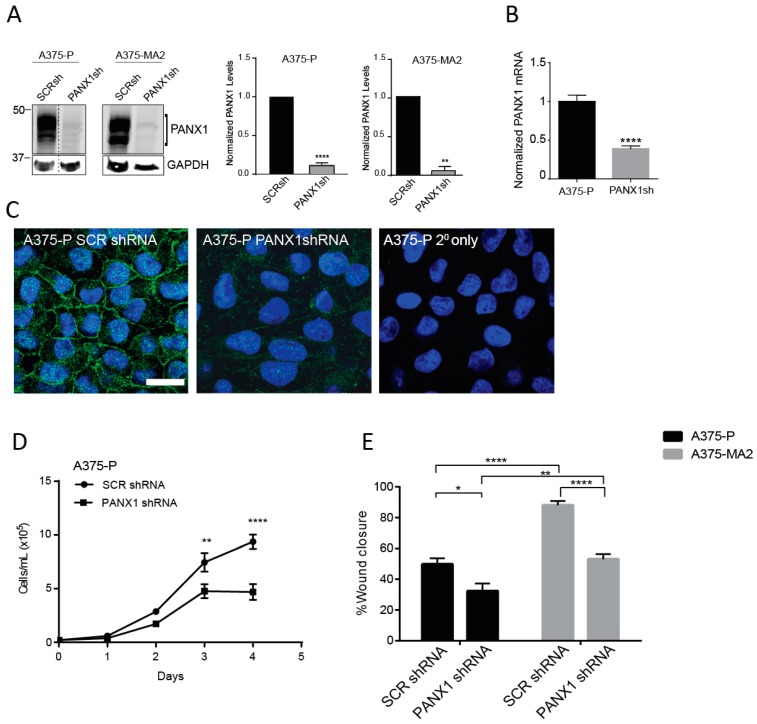
Knocking down PANX1 reduces growth and migration in human melanoma cells. (**A**) A375-P and A375-MA2 cells were transfected with either scrambled shRNA (SCRsh) as a control or shRNA constructs against PANX1 (PANX1sh). Western blot analysis confirmed PANX1 knockdown in both cell lines. GAPDH was used as loading control. Dotted line indicates extra lanes removed from the same blot scanned at the same intensity. Graphs depict the quantification of Western blots from three separate experiments conducted on different cell lysates, N = 3, student’s *t*-tests; **** *p* < 0.0001, ** *p* < 0.001 (**B**) PANX1 mRNA levels were measured by qPCR in control and PANX1 knockdown A375-P cells. Values in control samples were set to 1. Data are expressed as mean ± SEM, (N = 3, n = 9). Student’s *t*-test was used to analyse data; **** *p* < 0.0001. (**C**) A375-P cells transfected with scrambled shRNA (SCRshRNA, depicting PANX1 at the cell surface) or shRNA against PANX1 (PANX1shRNA, significantly reduced PANX1 signal) were fixed and stained with anti-PANX1 polyclonal antibodies (green). DNA was stained with Hoescht (blue). Staining with secondary antibody alone (2° only) was used as a control. Bar: 20 μm. (**D**) A375-P cells transfected with either scrambled shRNA (SCR shRNA) or shRNA against PANX1 (PANX1shRNA) were cultured at 20,000 cells per well in medium containing 10% FBS. Cells were counted daily up to 4 days after initial seeding of the cells onto the culture. There was a significant reduction in cell growth after three days in cells with reduced levels of PANX1. The data are expressed as mean ± SEM from four separate experiments conducted with three technical replicates. (N = 4, n = 12). Two-way ANOVA was used to statistically analyze the data. ** *p* < 0.01, **** *p* < 0.0001. ***E.*** Scratch assays were performed on a confluent monolayer of Scrambled (SCRshRNA) and PANX1 knockdown (PANX1shRNA) A375-P and A375-MA2 cells to assess migration in growth media with chFBS. The distance travelled by the cells represents cell migration three days following the initial scratch and treatment application. Data are expressed as mean ± SEM (N = 3, n = 9).

Previously, we have shown that the malignant properties of a mouse melanoma cell line were reduced following shRNA-induced silencing of Panx1 [17]. Here, we assessed the effect of shRNA knockdown of PANX1 on the cellular properties of two human melanoma cell lines that express endogenous PANX1 and have different aggressiveness profiles. Using scrambled shRNA as a control, we confirmed a significant PANX1 knockdown in A375-P and A375-MA2 melanoma cells by immunoblotting (Figure 4A). Quantitative RT-PCR revealed 62% (±0.03) reduction in the levels of PANX1 transcripts in A375-P cells transfected with shRNA against PANX1 (PANX1sh) compared to scrambled control (SCRsh) (Figure 4B). Immunofluorescence microscopy analysis showed a substantial reduction in PANX1 immunoreactivity in A375-P cells transfected with PANX1 shRNA compared to scrambled control (SCR shRNA) (Figure 4C).

We next investigated the proliferation rate of A375-P cells with PANX1 knockdown compared to scrambled control. Our data show that reduced PANX1 level in A375-P cells results in a significant two-fold decrease in proliferation of melanoma cells after four days in culture (Figure 4D). Results of scratch-wound assays revealed that A375-P and A375-MA2 cells with reduced levels of PANX1 exhibit 35% to 40% reduced migratory capacity compared to control cells (Figure 4E). Overall our results indicate a significant role for PANX1 in regulation of growth and migration of human melanoma cells.

### 2.5. PANX1 Channel Blockers Reduce the Tumorigenic Properties of Human Melanoma Cells In Vitro

Next, we evaluated the outcome of a pharmacological inhibition of PANX1 channels using PBN and CBX. These compounds were selected to block PANX1 channels due to their availability and their common use as *bona fide* Panx1 channel blockers [20,24,50,51]. Based on the doses reported in the literature and our results from cytotoxicity assays (Supplementary Figure S2A), 1 mM PBN or 100 μM CBX were selected for in vitro and xenograft experiments.

**Figure 5:**
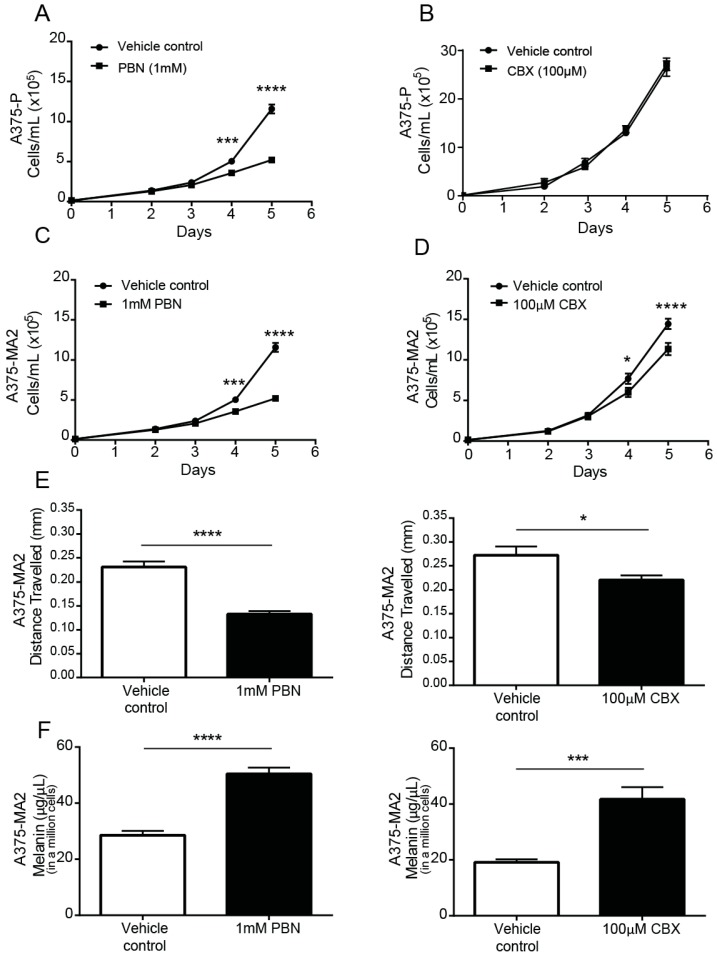
PANX1 blockers reduce the tumorigenic properties of human melanoma cells in vitro. A375-P melanoma cells were cultured at 20,000 cells per well in medium containing 10% FBS in the presence of PBN (1 mM) in (**A**) and CBX (100 µM) in (**B**). Similarly, A375-MA2 melanoma cells were cultured in the presence of PBN (**C**) or CBX (**D**) added to the culture medium. Cells were counted daily up to 4 days after initial seeding of the cells onto the culture. The data are expressed as mean ± SEM from three separate experiments conducted with three technical replicates. (N = 3, n = 9). (**E**) Scratch assays were performed on a confluent monolayer of A375-MA2 cells in the presence of PBN (1 mM, N = 3, n = 22) or CBX (100 µM, N = 3, n = 20) to assess migration. Data are expressed as mean ± SEM. Vehicle treated cells were used as control in the experiment. A significant decrease in A375-MA2 cell migration was noted in the presence of PBN and CBX. (**F**) Significantly more melanin was extracted from A375-MA2 cells treated with either 100 μM CBX (N = 3, n = 15) or 1 mM PBN (N = 3, n = 13) compared to vehicle controls. Statistical analyses for growth curves were performed using a two-way ANOVA followed by a Sidak test. Statistical analyses for migration assays and melanin extraction data includes student’s *t*-tests; * *p* < 0.05, *** *p* < 0.001, **** *p* < 0.0001; Bars indicate SEM.

Following a single dose of PBN or CBX in serum-containing medium, A375-P (Figure 5A,B) and A375-MA2 (Figure 5C,D) melanoma cell numbers were significantly lower than those in vehicle-treated cultures after 4 days of incubation with the drugs, except for CBX in A375-P cells (Figure 5B), which were not significantly affected. The doubling time of A375-MA2 cells was increased with both blockers (Supplementary Figure S2B). The A375-MA2 cell line was chosen for subsequent migration, melanin and chick-CAM test since it has been reported to be the most migratory and metastatic of the two and had the lowest level of pigment. Using a scratch-wound assay, we observed that the distance migrated three days following the initial scratch was significantly reduced when PBN or CBX was present in the medium of A375-MA2 cells (Figure 5E). To evaluate if there were changes in melanin synthesis in the presence of the PANX1 channel blockers, melanin was extracted from one million A375-MA2 cells cultured with either PBN, CBX or the vehicle control for three days. A375-MA2 cells produced 21.87 μg/μL more melanin when treated with PBN (Figure 5F). Similarly, after CBX treatment, A375-MA2 cells produced roughly double the amount of melanin (on average 22.59 μg/μL more melanin) than controls (Figure 5F). Taken together, these results suggest a reduction in growth and migration of human melanoma cells, with an increase in melanin production in the presence of PANX1 channels inhibitors.

### 2.6. Probenecid and Carbenoxolone Significantly Reduce A375-MA2 Tumor Growth of Human Melanoma Xenografts

**Figure 6:**
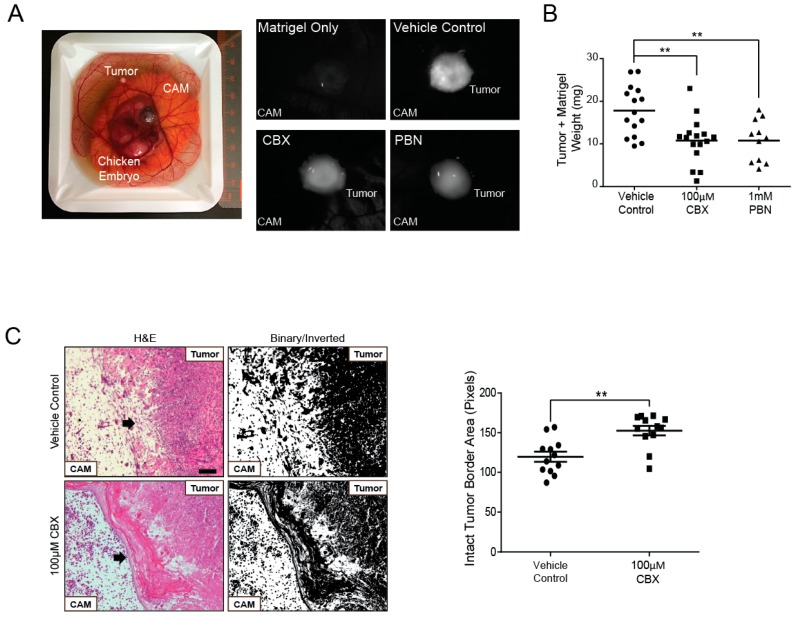
PANX1 blockers significantly reduced A375-MA2 melanoma tumor weight and invasion in chicken embryo xenografts. (**A**) One million A375-MA2 human melanoma cells were incubated with a DiO’lipophilic cell tracer, combined with Matrigel (1:1) and seeded onto the chorioallantoic membrane (CAM) of a ten-day-old chick embryo ex vivo. A375-MA2 tumors treated for one week with 100 μM CBX (N = 16) or 1 mM PBN (N = 11). (**B**) Tumors treated with CBX or PBN weighed significantly less than tumors treated with the vehicle control (N = 15). Statistical analyses were performed using one-way ANOVA followed by a Tukey’s post-hoc test. Lines represent the mean, ** *p* < 0.01. (**C**) A375-MA2 melanoma tumors were extracted from the CAM on day 18 of the experiment, sectioned and stained using H&E to analyze tumor structure. Tumors treated with the vehicle control (N = 4) showed an undefined border between the tumor and the CAM, possibly due to A375-MA2 cell invasion into the CAM. In contrast, tumors treated daily with 100 μM CBX (N = 4) were more easily removed from the CAM and displayed a defined tumor edge. Arrows indicate the edge of the tumors. To quantify CAM integrity, H&E images were converted to binary and inverted. A standard frame area was used in each image to measure the mean grey intensity in pixels as a measure of the area of intact CAM-tumor interface. The area of the intact tumor border was significantly increased in A375-MA2 tumors treated daily with 100 μM CBX for one week compared to those treated with the vehicle control (N = 4, n = 12). Scale bar: 50 μm and is the same for all images in (**C**). Bars indicate mean ± SEM. ** *p* < 0.01.

To evaluate changes in tumor growth when PANX1 channels are blocked, a chicken embryo xenograft model was utilized. A375-MA2 melanoma cells were pre-loaded with DiO lipophilic cell tracer for tumor visualization and seeded onto chick embryo chorioallantoic membranes (CAM). CBX, PBN, or a vehicle control was topically applied onto tumors daily following the initial inoculation for six days. Tumors were visible in each treatment condition and appeared to grow on the surface of the CAM (Figure 6A). When tumors were excised, we observed that tumors treated daily with either blocker weighed significantly less than those treated with the vehicle control (Figure 6B). We also conducted histological analysis of the tumors and their surrounding microenvironment and found that untreated tumors had irregular tumor edges with many cells appearing to invade into the CAM (Figure 6C). In contrast, tumors treated with CBX showed a well-defined border between the tumor and the CAM (Figure 6C). We measured the integrity of the tumor-CAM interface by obtaining inverted binary images using ImageJ based on [52]. The mean of the grey areas in pixels were calculated from a standard frame area that encapsulated the most superficial layer of the CAM. Quantification revealed that the area of the intact tumor border was significantly increased in A375-MA2 tumors treated with CBX compared to those treated with the vehicle control, suggesting CBX treatment of A375-MA2 cells decreased cell invasion into the CAM (Figure 6C, right panel). These results support a reduction in tumor growth and potentially a less invasive nature of these melanoma tumors in the presence of PANX1 channel blockers.

### 2.7. Inhibition of PANX1 Alters the Signaling Profile of Melanoma Cells

**Figure 7:**
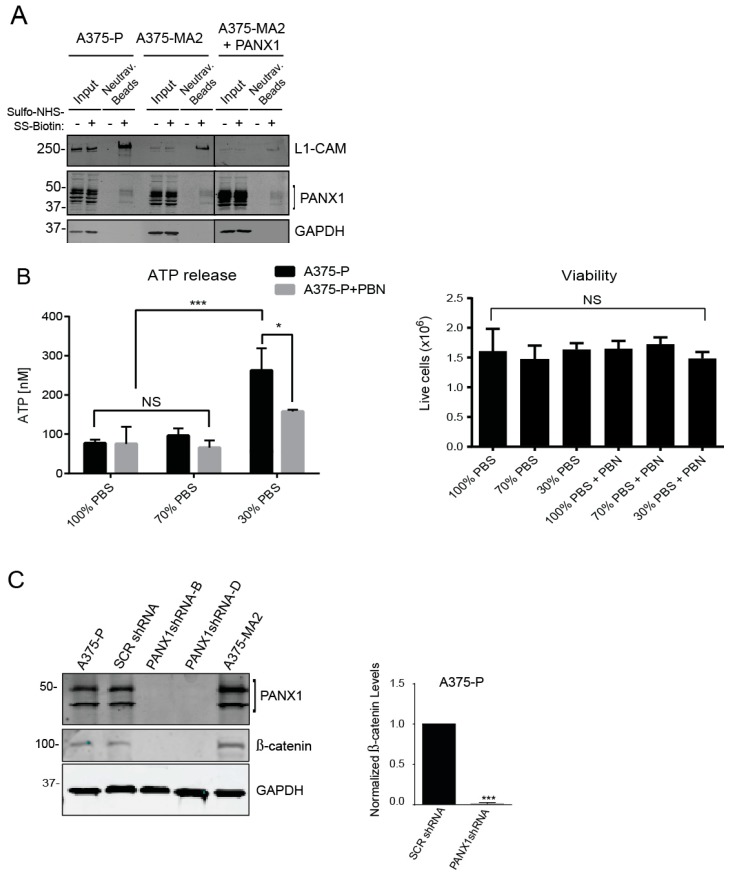
Inhibition of PANX1 alters the signaling profile of melanoma cells. (**A**) Protein lysates from A375-P, A375-MA2 cells with endogenous expression of PANX1 as well as A375-MA2 cells overexpressing PANX1 were used for cell-surface biotinylation assays. Negative controls were prepared in parallel in absence of biotin labeling reagent. Western blotting for L1-CAM and GAPDH were used as positive control of cell surface protein and control for non-specific biotinylation of intracellular/cytoplasmic proteins, respectively. Only a fraction of PANX1 is localized to the cell surface of cells examined in this experiment. (**B**) A375-P cells were washed in PBS and incubated with appropriate isotonic solution for 5 min (100% PBS (control), 70% PBS or 30% PBS) in the presence of 1 mM PBN. There was a significant decrease in ATP released into the culture medium in the presence of 1 mM PBN. Plotted values are means ±S.D. * *p* < 0.05, *** *p* < 0.0005 as determined by a two-way ANOVA with a Tukey’s multiple comparisons test. Viable cells were counted after each treatment and plotted in the right panel. There was no significant difference in the number of live cells in each treatment condition. (**C**) A375-P cells were transfected with either scrambled shRNA (SCRshRNA) as a control or two different shRNA construct against PANX1 (PANX1shRNA -B and -D). Cells were lysed, and equal amounts of protein were resolved by 12% SDS-PAGE. Western blot analysis using polyclonal antibodies confirmed PANX1 knockdown. GAPDH was used as loading control. There was a significant reduction in the abundance of β-catenin in PANX1 knockdown cells. Cell lysates of non-transfected A375-P and A375-MA2 cells were used as additional controls in the experiments. Graph depicts the quantification of western blots from three separate experiments, N = 3, student’s *t*-tests; *** *p* < 0.0001.

Although PANX1 has been shown to have a predominantly cell surface localization when exogenously expressed [45], we noticed a marked intracellular signal from PANX1 protein in immunofluorescence microscopy images of both patient-derived primary melanoma cells (Figure 2B) and established human melanoma cell lines (Figure 3A). Consistent with these observations, cell-surface biotinylation assays revealed that only a fraction of total PANX1 is present at the cell surface (Figure 7A) and intracellular PANX1 accounts for a major fraction of PANX1 immunoreactivity in A375-P and A375-MA2 cells. Of note, overexpression of PANX1 in A375-MA2 cells does not substantially increase the abundance of PANX1 at the cell surface (Figure 7A), suggestive of mechanisms by which melanoma cells may retain PANX1 intracellularly and prevent its translocation to the cell surface. As a positive control for cell-surface biotinylation, we used the surface protein L1-CAM, and as a negative control we used the intracellular protein GAPDH. These results suggest that the two different pools of PANX1 may have different functions at the cell surface versus intracellularly.

Cell surface-associated PANX1 channels mediate release of various substrates, including ATP [4]. ATP plays an essential role in the regulation of signals promoting melanoma cell migration and invasion [12,53]. Therefore, we investigated PANX1-mediated ATP release in A375-P cells stimulated with a hypotonic solution (30% PBS, 90 mOsm/kg) [54]. Blocking PANX1 function using PBN, significantly reduced the ability of A375-P cells to release ATP (Figure 7B). Cell viability assays showed comparable numbers of viable cells after all treatments (Figure 7B, right panel). Regarding the potential function of the intracellular pool, we have previously shown that shRNA-mediated Panx1 knockdown decreases β-catenin levels in a mouse melanoma cell line B16-BL6 [55]. Thus, we postulated that reducing PANX1 might similarly affect the Wnt/β-catenin pathway in human melanoma cells. Immunoblot analysis of lysates from melanoma cells transfected with scrambled shRNA (SCR shRNA) showed comparable endogenous levels of both β-catenin and PANX1 to un-transfected A375-P and A375-MA2 cells (Figure 7C). A substantial decrease in the abundance of β-catenin was observed in A375-P cells with knockdown of PANX1 using two different shRNA constructs (Figure 7C. Note different PANX1 banding pattern due to 12% gel). These data show a potential role for PANX1 in the regulation of the important Wnt/β-catenin signaling pathway.

## 3. Discussion

To evaluate the translational potential of our findings, we first evaluated the presence of PANX1 in human melanoma samples. Our in-silico analysis of TCGA and microarray expression databases indicated that although normal skin samples have a basal expression of PANX1, the transcript levels are significantly increased in primary melanoma tumors as shown on Figure 1A. However, once the expression of PANX1 is upregulated in primary melanoma, it does not seem to change significantly throughout the disease progression, from the T1 stage to advanced T3 stages (Figure 1B), although these conclusions would require analyses of larger sample numbers for confirmation.

In contrast with other studies that have reported marked differences in the expression of Panx1 between rat and human cancer cell lines [15], we found that human melanoma cells and biopsies were all positive for PANX1 regardless of the disease stage. This is consistent with reports of high PANX1 levels in other cancer types and in mouse melanomas [12], and would make it possible to target this protein in primary tumors as well as in advanced metastatic melanoma. However, at the mRNA and protein levels we observed variation among patient samples, which could be representative of the variable expression in human patients or due to the heterogeneity of the tumors from where the sample cores were obtained.

To complement our studies with primary tumor samples (Figure 1), we also isolated and obtained populations of MITF-positive primary melanoma cells from fresh patient-derived tumors and confirmed that they exhibited high levels of endogenous PANX1 protein. We cannot rule out that other cells in the stroma, as well as immune cells, and fibroblasts may also express PANX1. This was also true from established human melanoma cell lines that displayed high endogenous PANX1 levels, and similar to what was observed in the mouse B16 lines [17], the levels of expression were proportional to the aggressiveness of the line. Interestingly, PANX1 in all the cells examined displayed both an intracellular localization as well as cell surface labeling. This localization profile in melanoma cells is different from the predominant cell surface localization observed by immunostaining with the same antibodies when PANX1 is exogenously expressed in normal reference cells [45]. The two cell lines chosen for this study display different properties, as A375-MA2 is more aggressive and metastatic in vivo but grows slower in vitro compared to A375-P. However, both lines express endogenous PANX1 and had similar changes in cellular and tumorigenic properties upon PANX1 knockdown or blockade with CBX and PBN, indicating that inhibition of PANX1 function likely participate in the observed phenotypic changes.

Regardless of the subcellular localization of PANX1, the pharmacological blockers used in this study can act at the surface or permeate into the cell and block the channel function effectively, as has been previously reported in other systems [24,50]. Using pharmacological blockers to impair PANX1 channel function resulted in a significant reduction in the tumorigenic properties of the melanoma cells. The only exception was the lack of CBX effect on A375-P cell growth (Figure 5B), which could be potentially due to lower levels of PANX1 in these cells compared to A375-MA2s, or to differences in the stability or processing of the compounds in cell culture. Melanin production (a marker of melanocytic differentiation, [56]) was increased with all the treatments, indicating a potential effect of PANX1 channel function on the cell signaling of melanoma cells as we had previously reported for mouse melanomas [55].

It is possible that PANX1-associated ATP may act in conjunction with purinergic receptors like P2X7 (reviewed by [57]), to enhance melanoma growth [58]. This may occur through the release of ATP and other molecules that are secreted from melanoma cells to promote tumor growth and inflammation [59]. We tested ATP release as a potential mechanism and saw a significant reduction on ATP release from stimulated A375-P cells after treatment with PBN. However, as evidenced by the cell-surface biotinylation results, a subpopulation of endogenous PANX1 in these melanoma cells is localized intracellularly and may be playing a different role, unrelated to their canonical cell surface ATP release channel function. For example, the reduction in PANX1 may destabilize the Wnt/β-catenin complex and alter this important signaling pathway in melanoma [60,61]. The role of β-catenin in melanoma is complex and often paradoxical, as it appears to promote melanoma growth in early stages but repress late metastatic events, in what appears to be a canonical versus non canonical Wnt signaling effect (reviewed by Xue et al. [62]). However, it is clear that PANX1 and β-catenin are intimately connected since shRNA of pannexin 1 in either mouse [55] or human melanoma cells leads to a marked reduction in β-catenin levels. These findings are also consistent with recent reports published by Seref-Ferlengez et al., in which β-catenin levels were significantly reduced upon loading of mouse bones from Panx1^−/−^ mice compared to WT controls. In that study, they also observed that Panx1 modulates ATP signaling as well as Wnt/β-catenin signaling in a coordinated mechanism triggered by skeletal mechanical loading. [63]. Since β-catenin can be activated by pathways other than Wnt [64], it is important to consider the possibility that the Panx1/β-catenin interaction may occur in other signaling contexts. The two proteins may also be part of a scaffolding complex, as is the case for β-catenin and cadherins with important effects on cancer progression [65].

We complemented the pharmacological blocker tests with a genetic manipulation to silence PANX1 expression by shRNA and observed the same phenotypic changes with a reduction in cell proliferation and migration. Although knocking down the expression of PANX1 and closing the channel with blockers may not necessarily have identical effects (since the removal of the channel can also affect its interactome), we do observe a significant reduction in motility in vitro with blockers and shRNA. The observed reduction in migration, could be due in part to the fact that Panx1 can directly bind filamentous actin through the C-terminus [66]. Therefore, reducing the PANX1 levels could disturb its interaction with actin affecting migration. However, we observed a similar reduction on cell motility and migration when we close the channels with the blockers. This is consistent with our previous studies in mouse melanomas where Panx1 was found to be highly expressed in metastatic mouse melanoma cells compared to basal levels found in normal melanocytes [55]. In vitro tests had previously indicated that a knockdown of Panx1 in mouse melanomas reverted the cells to a more melanocytic-like phenotype with reduced tumorigenic properties [17]. In the xenografted tumors, the addition of PANX1 blockers not only affected tumor weight (and presumably growth), but also reduced the early stages of tumor migration and invasion, keeping the xenografted tumor borders intact and likely preventing the cancer cells from penetrating into the CAM. From our results, we conclude that PANX1 plays an important role in the regulation and progression of melanoma and may be a novel therapeutic target at all stages of melanoma progression, thus potentially improving current treatment options for individuals with melanoma.

## 4. Materials and Methods

### 4.1. In Silico Analysis of PANX1 Expression in Melanoma Tumors

Sample values from microarray study GSE15605 [42] using PANX1 probe identifier 204715_at was extracted from GEO2R (https://www.ncbi.nlm.nih.gov/geo/geo2r/). PANX1 mRNA expression z-scores (RNA Seq V2 RSEM) were generated using data in cBioPortal.org [43,44] from the Skin Cutaneous Melanoma Cohort generated by the TCGA Research Network (http://cancergenome.nih.gov/).

### 4.2. Cell Lines and Culture Conditions

Human melanoma cells lines A375-P (CRL-3224) and A375-MA2 (CRL-3223) [49], and Normal Rat Kidney (NRK, CRL-6509) cells were obtained from American Type Culture Collection (ATCC, Manassas, VA, USA). Cells were cultured in Dulbecco’s Modified Eagle Medium 1X (DMEM 1X) containing 4.5 g/L d-glucose, l-glutamine, 110 mg/L sodium pyruvate, 10% fetal bovine serum (FBS, Gibco, Thermo Fisher Scientific; Waltham, MA, USA), 100 units/mL penicillin, and 0.1 mg/mL streptomycin. All cells were incubated at 37 °C at 5% CO_2_. Trypsin (0.25%, 1 mM EDTA 1X; (Gibco, Thermo Fisher Scientific) was used to dissociate cells from culture dishes. Exogenous expression of human PANX1 in NRK cells was conducted as previously described [45], and used as a positive control.

### 4.3. Primary Melanoma Cells

All subjects gave their informed consent for inclusion before they participated in the study. The study was conducted in accordance with the Declaration of Helsinki, and the protocol was approved by the Ethics Committee of Western University and London Health Sciences Centre (HSREB#103381). Melanoma tumor biopsies obtained after informed consent from patients and processed according to the ethical protocol, were dissected by a pathologist immediately after surgery and placed on ice in a tube containing Dulbecco’s Phosphate Buffered Saline with calcium and magnesium chloride (D-PBS) and 10% FBS. Samples were washed with PBS, minced and transferred to a tube containing a collagenase digest solution (Krebs-Ringer bicarbonate buffer (Sigma Aldrich, St Louis, MO, USA), 2% BSA, 2 mg/mL collagenase (Worthington Biochemical Corporation, Lakewood, NJ, USA) and digested for 30 min at 37 °C. The tissue was separated in this mixture using sequentially smaller serological pipettes. The final suspension was filtered through a 100 μm cell strainer and centrifuged at 800× *g* for 10 min. The supernatant was removed by aspiration, and cell pellets were re-suspended in DMEM containing 10% FBS, 1 mL non-essential amino acids (Life Technologies), 2 mL MEM 2X vitamin solution (Gibco, Thermo Fisher Scientific) and 1% penicillin streptomycin. Cells were plated and cultured for 3 days at 37 °C at 5% CO_2_ to allow blood cells to separate from melanoma cells before medium was replaced. Primary cells at first passage (P1) were either processed for immunocytochemistry or lysed for protein extraction.

### 4.4. Protein Extraction and Immunoblotting

Frozen melanoma tumor biopsies (six primary melanomas, eight nodal metastases, four distant metastases) were obtained from the Ontario Institute for Cancer Research (Toronto, ON, Canada; OICR, HSREB#103381). Protein lysates were prepared with 1% Triton X-100, 150 mM NaCl, 10 mM Tris, 1 mM EDTA, 1 mM EGTA, 0.5% NP-40. Human cell lines and primary cell lysates were extracted using RIPA buffer (50 mM Tris-HCl pH 8.0, 150 mM NaCl, 1% NP-40 (Igepal) (Honeywell Fluka, Seelze, Germany), 0.5% sodium deoxycholate). Each buffer contained 1 mM sodium fluoride, 1 mM sodium orthovanadate, and one tablet of complete-mini EDTA-free protease inhibitor (Roche, Mannheim, Germany). Protein content in lysates was quantified using the bicinchoninic acid (BCA) assay (Thermo Fisher Scientific). Protein lysates (50 μg) were resolved by denaturing gel electrophoresis (10% SDS-PAGE) and transferred onto nitrocellulose membranes using an iBlot^TM^ System (Invitrogen). Membranes were blocked with 3% bovine serum albumin (BSA) with 0.05% Tween-20 in 1X phosphate buffer saline (PBS), and incubated with anti-human PANX1 antibody (1:1000; PANX1 CT-412; 0.35 μg/μL) [45]. Other antibodies used were mouse monoclonal anti-L1-CAM (clone CD171) cat#MA5-14140 (1:1000, Invitrogen), mouse anti-beta-catenin Cat# 610154 (1:1000, BD Biosciences, San Jose, CA, USA). Loading controls were obtained with an anti-glyceraldehyde 3-phosphodehydrogenase (GAPDH) antibody (1:1000; Millipore Cat# MAB374, RRID: AB_2107445). Peptide pre-absorption assays were performed by incubating the diluted PANX1 antibody with 50:1 molar excess of its corresponding human PANX1-peptide (6 μg/μL) for 30 min at room temperature before probing [45]. For detection, IRDye^®^-800CW and -680RD (LI-COR Biosciences, Lincoln, NE, USA) were used as secondary antibodies at 1:10,000 dilutions, and imaged using a LI-COR Odyssey infrared imaging system (LI-COR Biosciences)

### 4.5. Cell Surface Biotinylation Assays

Cells used for biotinylation were cultured until confluence in 60-mm culture plates and the cell monolayer was rinsed twice with ice-cold D-PBS. Proteins at the cell surface were labeled with a solution of 1.5 mg/mL EZ-Link™ Sulfo-NHS-SS-Biotin (Thermo Scientific) in D-PBS for 30 min on ice and protected from light. Negative controls were prepared in parallel in the absence of biotin labeling reagent. Then after one wash with D-PBS, 100 mM glycine in D-PBS was added to quench any remaining labeling biotin and incubated on ice for 30 min. Protein lysates were prepared as described previously and to pull-down the cell-surface-biotinylated proteins, 250 µg of total protein were incubated for 16 h with 50 µL (50% slurry) NeutrAvidin agarose beads (Thermo Scientific) in a final volume of 250 µL in D-PBS. The beads were then centrifuged (×500 *g*, 4 °C), washed three times with lysis buffer and resuspended in 2X Laemmli buffer containing 10% β-mercaptoethanol. To elute the bound protein fraction, the beads were placed at 95 °C in a heat block for 5 min, centrifuged and the supernatant was collected. Approximately 10 µg of total protein of lysates was used as input and 25 µL of bead supernatant were resolved in parallel in 10% SDS-PAGE gels and then transferred to nitrocellulose membrane. Immunoblotting for L1-CAM and GAPDH were used as positive control of cell surface protein and control for non-specific biotinylation of intracellular/cytoplasmic proteins, respectively.

### 4.6. ATP Release Assays

Tissue culture plates were coated overnight with 0.01% poly-L-lysine (Millipore Sigma, Burlington, MA, USA) A375-P cells (ATCC) were plated at 200,000 cells per well in a 24 well plate and allowed to attach overnight. All washes and media changes were performed gently with a P1000 pipette to avoid cell perturbations due to the force of vacuum aspiration. Cells were rinsed with serum-free DMEM (Thermo Fisher Scientific) and incubated for 15 min at 37 °C. The treatment group was incubated with 1 mM Probenecid (Thermo Fisher Scientific) at this time. To stimulate ATP release, cells were rinsed with PBS and incubated with 200 μL of the appropriate solution for 5 min (100% PBS (control), 70% PBS or 30% PBS). Treatment groups included 1 mM Probenecid under hypotonic conditions. Following the hypotonic incubation, 150 μL of the medium was collected, immediately placed on ice and centrifuged at 4 °C for 5 min at 800× g. DMEM + 10% FBS, 1% Penicillin/Streptomycin was added to wells to allow cells to recover at 37 °C and 5% CO_2_ during ATP measurements. Samples were processed for ATP content using the ATP Determination Kit (Thermo Fisher Scientific). According to the manufacturer’s protocol, 30 μL of supernatant was transferred to a 96-well white plate and 270 μL of reaction buffer was added. ATP content was determined quantitatively by luminometry, and the ATP concentrations were normalized to an ATP standard curve. Following ATP measurements, cells were trypsinized and counted with trypan blue to ensure no variability in cell viability between groups.

### 4.7. Immunohistochemistry

Patient-derived melanoma sections of 14 primary tumors, 14 nodal tumors, and 6 distant metastases as well as pre-imaged, sequentially sectioned tumors stained using H&E were provided by the Ontario Tumor Bank of the Ontario Institute of Cancer Research (OICR, ethically collected after informed consent). All samples handled according to our ethical protocol HSREB#103381 (Western University and LHSC). Paraffin embedded sections were deparaffinized using xylene and sequential dilutions of ethanol. Antigen retrieval was performed in 1.5% of Vector Labs Antigen Unmasking Solution (Vector Laboratories, Burlington, ON, Canada) and heating in a microwave at 80% power. Sections were labeled for PANX1 (1:50; PANX1 CT-412) and a peptide pre-adsorption assay was performed as previously described [45]. A rabbit anti-MITF antibody (1:100; Abcam, Cambridge, UK) was used to identify melanoma cells. Alexa Fluor 488 goat anti-rabbit IgG (2 mg/mL; 1:400) was used as a secondary antibody and Hoechst 33342 (1:1000 in water) (Thermo Fisher Scientific) was used to label cell nuclei. Slides were mounted using VECTASHIELD^®^ mounting medium (Vector Laboratories) before imaging.

### 4.8. Immunocytochemistry

Cells were cultured to 70–80% confluency on glass coverslips, washed with PBS, and fixed with ice-cold 80% (v/v) methanol and 20% (v/v) acetone for 15 min at 4 °C, and then blocked with 2% BSA-PBS. The same antibodies described previously were applied (PANX1 1:250, MITF 1:250, Hoechst 33342 1:1000). Coverslips were mounted using Airvol (Mowiol 4-88; Sigma Aldrich) prior to imaging.

### 4.9. Microscopy Analyses

For migration assays, a Zeiss Axiovert A1 brightfield microscope (Carl Zeiss Inc., Oberkochen, Germany) was used for imaging cell monolayers. For chick-CAM assays, fluorescently labeled A375-MA2 tumors were imaged using a Zeiss Lumar.V12 stereoscope (Carl Zeiss Inc.) with a GFP filter and a 0.8X NeoLumar objective. Immunofluorescence images of cells and tumor sections were obtained using a Zeiss LSM 800 Laser Scanning Fluorescence Confocal Microscope. The laser lines used include 405 nm (Hoechst 33342), 488 nm (Alexa488), and 561 nm (Alexa555). The tiling module of Zen2.3 was used to image and stitch together large tumor sections.

### 4.10. shRNA Knockdown of PANX1

A375-P and A375-MA2 cells were transfected with two constructs (PANX1 shRNA-B and PANX1 shRNA-D) from Origene (Rockville, MD, USA): PANX1 human 29-mer shRNA kit in pRS vector (TR302694) plus a non-effective 29-mer scrambled shRNA cassette as a control. shRNA-expressing cells were selected with puromycin, and two constructs (B and D) were examined for Panx1 knockdown (KD). Both constructs showed 80–90% reduction in PANX1 expression 7 days after transfection in melanoma cell lines. Cells were maintained under puromycin selection pressure and periodically examined for concomitant free-GFP expression and effective Panx1 knockdown by immunoblot. Experiments were conducted after verifying at least an 85% knockdown of Panx1 protein levels by Western blot.

### 4.11. Pannexin 1 Channel Blockers

Carbenoxolone disodium salt (≥98%; Sigma Aldrich) and water-soluble Probenecid (77 mg/mL; Thermo Fisher Scientific) were dissolved in Hanks’s Balanced Salt Solution (HBSS 1X, Gibco, Thermo Fisher Scientific); with calcium chloride, magnesium chloride, magnesium sulfate) to develop stock solutions of each compound. For in vitro assays, the blockers were added to culture medium only once, on day 0, whereas for chick-CAM assays, the blockers were applied once daily for 7 days. Final concentrations of 100 μM CBX or 1mM PBN were used for each assay.

### 4.12. WST-1 Cytotoxicity Assay

Cell Proliferation Reagent WST-1 (Sigma Aldrich) was used to assess cytotoxic effect of CBX and PBN on A375-MA2 cells according to manufacturer instructions with increasing concentrations of CBX, PBN, or HBSS vehicle control. Measurements at 450 nm and at a 690 nm were taken on an Epoch microplate spectrometer (Biotek, Winooski, VT, USA).

### 4.13. Growth Curves

In a 6-well culture plate, 20,000 A375-MA2 or A375-P cells were seeded in each well at day 0 of the experiment. Serum-containing media with a final concentration of 1 mM PBN, 100 μM CBX, or HBSS vehicle control was added to individual wells. For 4 days starting at day 2, cell numbers were determined using a Countess automated cell counter (Thermo Fisher Scientific) [17].

### 4.14. Migration Assays

A scratch assay was performed on A375-MA2 cells grown to confluency. Complete media with charcoal stripped FBS (chFBS) was prepared to remove the effect of growth factors and prevent proliferation. Media containing chFBS with either 100 μM CBX, 1 mM PBN, or HBSS vehicle control was added to the cells at the beginning of the experiment. Three pictures of individual squares on the gridded plate were taken immediately after initial scratch and 72 h later using a Zeiss Axiovert A1 brightfield microscope. The distance travelled (in the x-axis) was determined by dividing the total area the cells migrated by length of the square grid on the plate (2mm) using ImageJ software [17].

### 4.15. Melanin Extraction

In a 60 mm culture dish, 4 × 10^5^ A375-MA2 or A375-P human melanoma cells were seeded in complete medium containing 100 μM CBX, 1 mM PBN or HBSS vehicle control. Three days later, melanin was extracted from one million cells with 1M NaOH and 10% DMSO as described previously [17]. Melanin standard curves were developed using purified melanin (Sigma Aldrich). Absorbance was measured at 450 nm using an Epoch microplate spectrometer (Biotek).

### 4.16. Xenograft Tumor Growth in the Chick Chorioallantoic Membrane Assay (Chick-CAM Assay)

Chick-CAM assays were performed as described previously [17,67]. Briefly, Fertilized chicken eggs (McKinley Hatchery, St. Mary’s, ON, Canada) were incubated in a rotating incubator for 3 days. Embryos were transferred into weigh boats and incubated for 7 more days. DiOC_18_(3)(3,3′-Dioctadecyloxacarbocyanin Perchlorate) Lipophilic Cell Tracer (1:500) was used to label A375-MA2 cells. At day 10, 10^6^ A375-MA2 cells in serum-free media containing a PANX1 channel blocker or HBSS vehicle control were combined with 20 μL of Matrigel (1:1) and implanted onto the CAM. Tumors were treated daily with a topical application of blocker or vehicle. At day 17, tumors were imaged, excised and weighed [17,67]. For histological analysis, tumors were excised on day 18 of the experiment, retaining the surrounding CAM microenvironment, fixed in 10% formalin and embedded in paraffin as previously described [68]. Controls containing Matrigel-only were processed in parallel.

### 4.17. Hematoxylin and Eosin (H&E) Staining

Histology analyses were performed on A375-MA2 tumors from chick-CAM experiments. Adjacent tissue sections (7 μm) were deparaffinized, rehydrated by immersion in an ethanol gradient and stained with hematoxylin (Lerner Laboratories, VWR, Radnor, PA, USA) for 5 min. Sections were rinsed and then stained with eosin-Y (Lerner Laboratories) for 5 min. Samples were consecutively washed with ethanol and xylene and then mounted using Permount Mounting Medium (Electron Microscopy Sciences, Hatfield, PA, USA). Stained samples were imaged on a Leica DMIL LED brightfield, inverted microscope using a 20× magnification.

### 4.18. Tumor-CAM Interface Quantification

Histological images were converted to binary and inverted using ImageJ (National Institutes of Health (NIH), Bethesda, MD, USA) with a method that was designed based on other published ImageJ plugins used for yeast and cancer colony quantification [52]. As such, hematoxylin and eosin staining appeared black and blank space or areas of non-staining appeared white on the images. A standard frame area of 29.19 μm by 204.32 μm was positioned on each image to encapsulate the chorionic epithelium—the most superficial CAM layer—or what was left of it (N = 4, n = 12 for both vehicle control and CBX-treated tumors). The mean grey value within each standard area—which represents the densitometry value (in pixels) of the selected area—was calculated using ImageJ. Multiple measurements were taken along the CAM-tumor interface of each image to create an image average. This technique was used as a measure of tumor border integrity, where the higher numbers of black pixels (protein and cell staining) within the frame, corresponded to more intact CAM-tumor interfaces.

### 4.19. Statistical Analysis

All statistical analyses were performed using the statistical package of GraphPad Prism 6^®^ (San Diego, CA, USA). Unpaired Student’s *t*-tests were used for mean comparisons in migration assays, melanin and tumor-CAM interface quantification. One-way ANOVA followed by a Tukey test were applied for analysis of protein in cell lines, patient samples, cell viability and chick-CAM experiments. Two-way ANOVA followed by a Sidak or Tukey’s test were conducted for analysis of growth curves and ATP release. Results are expressed as the mean ± SEM of at least three independent experiments.

## 5. Conclusions

We concluded that human melanomas express high levels of PANX1 that when targeted via chemical blockers or genetic silencing reduce melanoma growth, migratory capacity, tumorigenesis and invasiveness. Inhibiting PANX1 channels increases melanin production, and affects signaling pathways that regulate melanoma progression, identifying PANX1 as a potential therapeutic target for melanoma treatment.

## Supplementary Materials

The following are available online at http://www.mdpi.com/2072-6694/11/1/102/s1, Figure S1: Immunofluorescence of PANX1 expression in representative patient-derived primary melanoma tumors. Figure S2: WST-1 cytotoxicity assay used to assess A375-MA2 cell viability when CBX or PBN is applied.
