# Supplementary material for: Inhibition of Pannexin 1 Reduces the Tumorigenic Properties of Human Melanoma Cells
Source: Cancers (Basel). 2019 Jan 16;11(1):102. doi: 10.3390/cancers11010102 (PMC6356688; doi:10.3390/cancers11010102)
Supplement: Supplementary file 1 [file cancers-11-00102-s001.pdf]

cancers

MDPI

Inhibition of Pannexin 1 Reduces the Tumorigenic Properties of Human Melanoma Cells

Taylor J. Freeman, Samar Sayedyahossein, Danielle Johnston, Rafael E. Sanchez-Pupo, Brooke O'Donnell, Kenneth Huang, Zameena Lakhani, Daniel Nouri- Nejad, Kevin J. Barr, Luke Harland, Steven Latosinsky, Aaron Grant, Lina Dagnino and Silvia Penuela

Supplementary Material

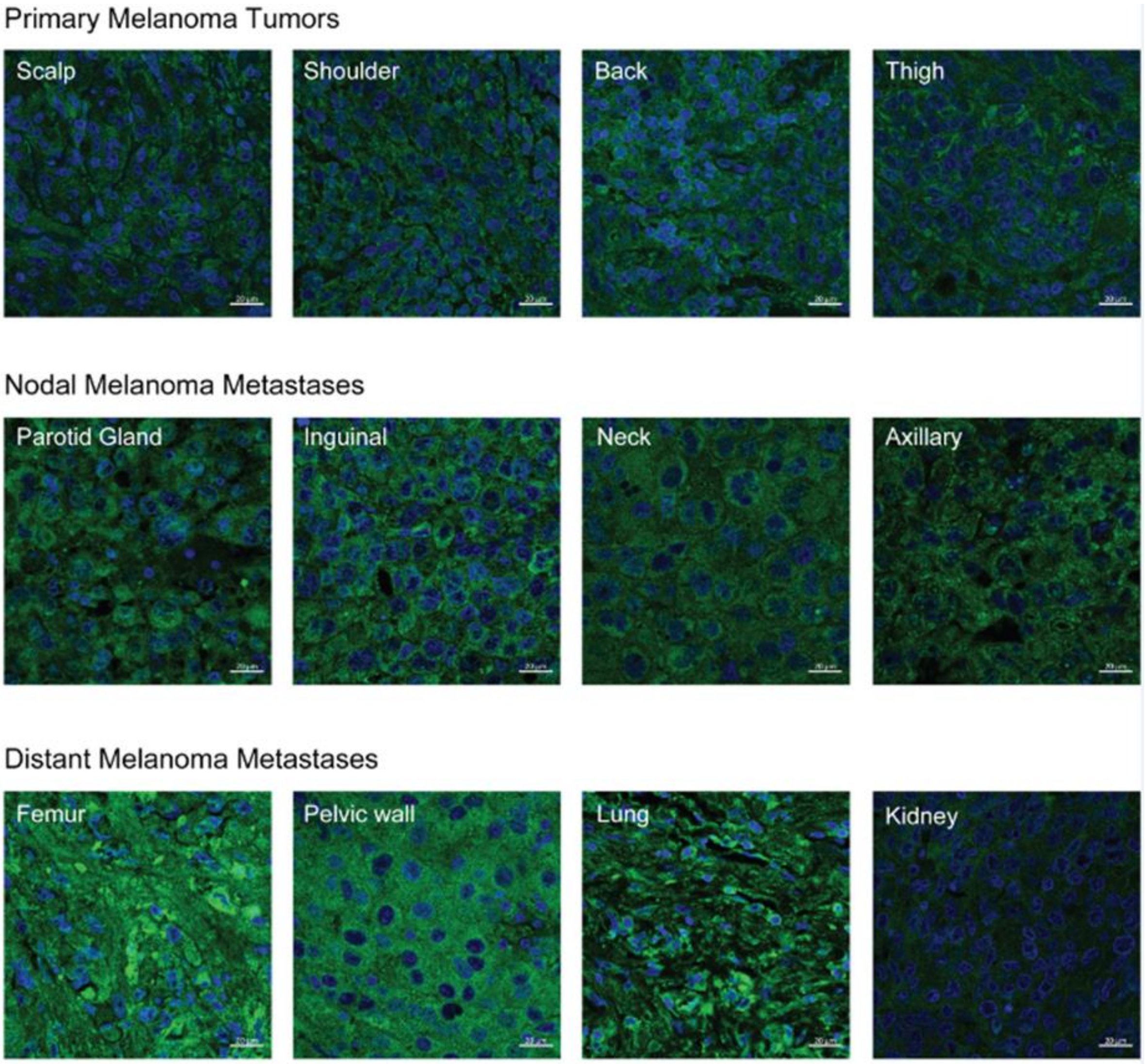

Supplementary Figure 1. Immunofluorescence of PANX1 expression in representative patient-derived primary melanoma tumors, nodal and distant melanoma sections from different tumor locations provided by OICR. Each panel represents a different patient. PANX1: green, Hoechst: blue; Scale: 20µm.

A

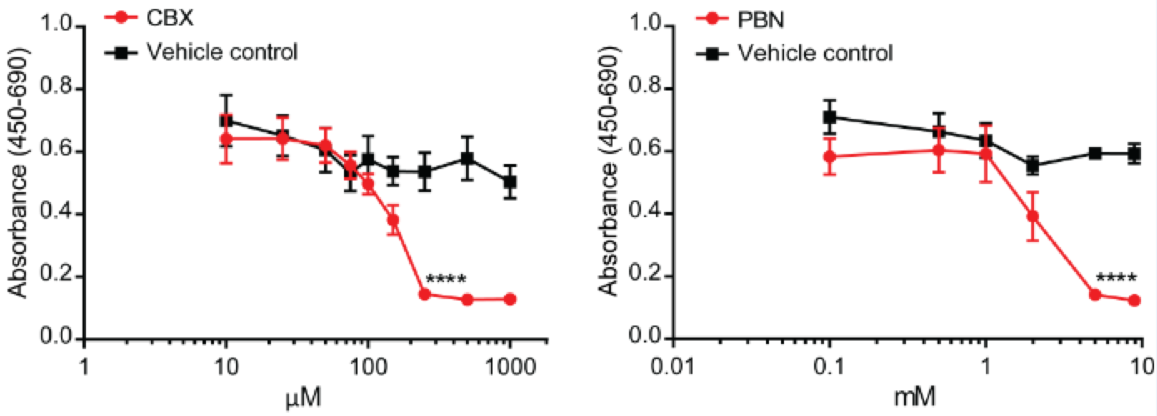

B

A375-MA2

Condition	Doubling Time (hours±SD)
Vehicle Control (CBX)	18.05 ± 0.24
100μM CBX	19.05 ± 0.34
Vehicle Control (PBN)	19.10 ± 0.21
1mM PBN	22.91 ± 0.54

Supplementary Figure 2. (**A**) A WST-1 cytotoxicity assay was used to assess A375-MA2 cell viability when CBX or PBN is applied. Cytotoxic effects do not occur at 100μM CBX and 1mM PBN indicating that results from *in vitro* and *in vivo* experimental assays are due to channel blockade rather than a decrease in cell viability. Significant cytotoxic effects occur at 250μM CBX or 5mM PBN in A375-MA2 melanoma cells. Statistical analyses for WST-1 assays were performed using a two-way ANOVA with multiple comparisons followed by a Sidak test. (**B**) Doubling times of A375-MA2 cells increased when 100μM CBX (N = 4, n = 12) or 1mM PBN (N = 3, n = 9) was applied to cells in comparison to vehicle control. Data for doubling time was derived from the curves in Figure 2A using a nonlinear regression for exponential growth. **** $p < 0.0001$; Bars indicate S.E.M.
